# Hsp27 as a marker of cell damage in children on chronic dialysis

**DOI:** 10.1007/s12192-012-0339-1

**Published:** 2012-04-22

**Authors:** Kinga Musiał, Danuta Zwolińska

**Affiliations:** Department of Pediatric Nephrology, Wrocław Medical University, Borowska 213, 50-556 Wrocław, Poland

**Keywords:** Hemodialysis, Peritoneal dialysis, sFas, sFasL, MMP-7, TIMP-1

## Abstract

Intracellular heat shock protein (Hsp) 27 is a potent anti-apoptotic factor that, among other activities, prevents the binding of membrane receptor Fas to its ligand FasL. However, the potential role of extracellular Hsp27 and possibilities to control it have not been clarified. Moreover, there are no data on relations between Hsp27, sFas/sFasL system, matrix metalloproteinases (MMPs) and their tissue inhibitors (TIMPs) in patients with chronic kidney disease (CKD)—neither children nor adults. The aim of this study was to evaluate serum concentrations of Hsp27 and their potential regulators (sFas, sFasL, MMP-7, TIMP-1) in children with CKD and on chronic dialysis. Twenty-six CKD children stage 5 still on conservative treatment, 19 patients on hemodialysis (HD), 22 children on automated peritoneal dialysis (APD), and 30 controls were examined. Serum concentrations of Hsp27, sFas, sFasL, MMP-7, and TIMP-1 were assessed by ELISA. Median values of Hsp27 were significantly elevated in all dialyzed patients vs. those in pre-dialysis period and vs. controls, the highest values being observed in subjects on HD. Regression analysis revealed that MMP-7, TIMP-1, sFas, and sFasL were the best predictors of Hsp27 concentrations in dialyzed patients. Children with CKD are prone to Hsp27 dysfunction, aggravated by the dialysis commencement, and more pronounced in patients on hemodialysis. Correlations between Hsp27 and examined parameters suggest the potential role for Hsp27 as a marker of cell damage in the pediatric population on chronic dialysis.

## Introduction

Chronic inflammation, immune system activation, and enhanced apoptosis are characteristic features of chronic kidney disease (CKD), additionally aggravated by the dialysis procedure and the membrane type (Andreoli et al. 2007; Soriano et al. 2005). Various heat shock proteins take part in the abovementioned processes. Among them, intracellular Hsp27 seems to play a crucial role in apoptosis regulation, controlling both intrinsic and extrinsic pathways of programmed cell death (Concannon et al. 2003; Sanchez-Nino et al. 2012). The extrinsic one is initiated by the binding of membrane receptor Fas to its ligand FasL. This action can be inhibited by Hsp27, blocking the Daxx translocation to the membrane and its interaction with Fas (Nagata and Goldstein 1995; Charette et al. 2000). Hsp27 can also regulate negatively apoptosis by both upstream and downstream inhibition of cytochrome c release (Concannon et al. 2003). However, there are no data on the character of Hsp27/Fas/FasL intracellular interactions in the course of chronic kidney disease or under dialysis conditions.

Recent investigation has focused on the soluble forms of abovementioned parameters, sHsp27, sFas, FasL, and their potential role in cell damage. Extracellular Hsp are mainly considered the markers of damage, released under pathological conditions by the injured or necrotic cells, and “danger signals,” directing the innate immune system towards Hsp recognition by antigen presenting cells (APC) (Calderwood et al. 2007; Joly et al. 2010). The subsequent production of pro-inflammatory cytokines by APC and activation of nuclear factor (NF)-κB facilitates adaptive immune response and antigen presentation to cytotoxic T cells (Srivastava 2002). However, the significance of this sequence has been questioned recently by van Eden et al. (van Eden et al. 2012). The authors suggested that Hsp should not be treated solely as damage-associated molecular patterns (DAMPs), responsible mainly for pro-inflammatory stimulation of the immune system, but also as factors able to suppress immune response and favor the process of tissue restoration after damage. Henderson and Pockley went even further, hypothesizing that extracellular Hsp may play actively as autocrine, paracrine, or endocrine signals, either cardioprotective (like Hsp27) or cardiopathologic (Henderson and Pockley 2012). Such interpretation surely throws a new light on the role of Hsp in CKD-related complications.

Uremic toxicity increases Fas and FasL expression on cells, thus evoking changes in sFas and sFasL concentrations (Jaber et al. 2001). Apart from their anti-apoptotic (sFas) and pro-apoptotic (sFasL) activity, they are regarded as markers of inflammation and endothelial dysfunction, and therefore classified as hallmarks of atherosclerosis (El-Agroudy and El-Baz 2010; Dalboni et al. 2003). However, there are no data on potential links between extracellular Hsp27 and sFas/sFasL system in the course of CKD.

Other inflammation markers, like hsCRP or IL-4, have not been tested for their potential relations with Hsp27 either. E-selectin, adhesion molecule expressed on endothelial cells, constitutes an ideal marker of their activation, which is followed by sE-selectin cleavage, pointing at endothelial damage. However, there are no data on connections between this molecule in either of the forms and intra- or extracellular Hsp27.

Connections of the abovementioned markers with extracellular proteolytic enzymes, matrix metalloproteinases (MMPs) and their tissue inhibitors (TIMPs), have been known in the case of sFas/sFasL. sFasL is proteolytically shed from its membrane-bound form by MMPs (Kayagaki et al. 1995). The strongest evidence for such activity concerns matrilysin (MMP-7) and has been shown in in vitro experiments (Vargo-Gogola et al. 2002). It has also been suggested that other MMPs/TIMPs may control the sFas/sFasL activity (Musiał and Zwolińska 2011). Nothing is known about possible connections between extracellular Hsp27, MMPs, and TIMPs in chronic kidney disease patients.

Therefore, our aim was to assess the serum levels of Hsp27 in relation to sFas and sFasL, in children with chronic kidney disease, in patients on peritoneal dialysis and hemodialysis, searching for the differences between those modalities. We also investigated the correlations between Hsp27, matrix metalloproteinases (MMP-7), their tissue inhibitors (TIMP-1), markers of inflammation (high sensitivity CRP, IL-4), and endothelial damage (sE-selectin) in the pediatric population with CKD.

## Materials and methods

Sixty-seven CKD patients enrolled in the study were divided into three groups. The first group (CKD) contained 26 patients (ten girls, 16 boys) aged 2.0–16.5 years (median, 10.5 years) with CKD stage 5, yet on conservative treatment (median glomerular filtration rate (GFR) calculated according to the Schwartz formula 13 ml/min/1.73 m^2^) (Schwartz et al. 2009). Primary diseases causing CKD were reflux nephropathy (13), chronic glomerulonephritis (nine), neurogenic bladder (three), hemolytic uremic syndrome (one). In all patients, phosphate binders and vitamin D metabolites were supplemented.

The second group consisted of 22 children (12 girls, ten boys) aged 4.0–15.5 years (median, 10.0 years) on automated peritoneal dialysis (APD, Baxter, Home choice), five of them having residual renal function. Mean Kt/V value was 1.4. The patients had five to eight exchanges of dialysis fluid during the night and, if necessary, one or two during the day. Peritoneal fluids used in our patients had glucose concentrations of 1.36 % or 2.27 %. The causative factors in CKD were chronic pyelonephritis (seven), chronic glomerulonephritis (six), polycystic kidney disease (two), neurogenic bladder (three), hemolytic uremic syndrome (two), and unknown (two).

The third group included 19 patients (ten girls, nine boys) aged 10.5–17.0 years (median, 13.5 years), hemodialyzed on polysulfone membranes, only three out of them with residual kidney function. Hemodialysis (HD) sessions (3.5–4 h) were performed three times a week, using bicarbonate dialysate, the blood flow ranged from 150 to 200 ml/min, dialysate flow did not exceed 500 ml/min. The membrane area was between 1.0 and 1.6 m^2^, the dialyzers were not reused. The water, purified by reosmosis, was regularly checked for contamination. Mean Kt/V value was 1.3. All patients were on stable anticoagulation regimen using non-fractionated or low-molecular weight heparin. The causative factors in chronic renal failure were chronic glomerulonephritis (seven cases), chronic pyelonephritis (seven), neurogenic bladder (two), polycystic kidney disease (one), and unknown (two).

Thirty children (16 girls, 14 boys) aged 5.5–15.5 years (median, 10.0 years) with primary nocturnal enuresis and normal kidney function, served as controls.

None of the patients showed clinical evidence of infection, had diabetes, malignancies or vasculitides, smoked, took antibiotics, statins, corticosteroids, or immunosuppressive therapy. They were also free of such comorbidities as diabetes, cardiovascular disease, peripheral vascular disease, or obesity. In the CKD group, blood pressure was well controlled either without medication (18 children) or with the use of calcium channel blockers (five patients) and β-blockers (three children). All APD children had their blood pressure values below the 90th percentile, adjusted for gender and age, according to the criteria of the fourth report on high blood pressure in children and adolescents (National 2004) and did not require anti-hypertensives. The blood pressure in our HD patients was within normal values without medication (12 patients) or was well controlled with the use of calcium channel blockers only (three) or calcium channel blockers with β-blockers (four).

Informed consent was obtained from the subjects and their parents, if necessary. The research project has been approved by the university ethics committee in accordance with the Helsinki declaration.

Blood samples were drawn after an overnight fast from peripheral veins in CKD and APD patients and in controls, in HD subjects twice—from the afferent line of the first-use dialyzer before starting an HD session and after finishing it. Samples were clotted for 30 min, centrifuged at 4°C for 10 min, and then serum was stored at −20°C until assayed.

Serum concentrations of Hsp27, sFas, sFasL, MMP-7 (matrilysin), and TIMP-1 were evaluated by commercially available ELISA kits (Stressgen, R&D Systems, Abingdon, UK). Standards and serum samples were transferred to 96-well microplates pre-coated with recombinant antibodies to human Hsp27, sFas, sFasL, MMP-7, and TIMP-1. Each sample was tested in duplicate, and the arithmetical mean was considered a final result. Measurements were performed according to the manufacturer’s instructions; results were calculated by reference to standard curves.

In all patients, high sensitivity CRP (nephelometry by Dade Behring, Marburg, Germany), interleukin (IL)-4 and sE-selectin (ELISA kits by R&D Systems) as markers of inflammation/endothelial dysfunction, were also evaluated.

### Statistical analysis

Results are expressed as median values and interquartile ranges. Since the null hypothesis of normality of distribution was rejected by Shapiro–Wilk test, multiple comparisons and comparisons in pairs were evaluated by using nonparametric tests (Kruskall–Wallis, Mann–Whitney *U*, Wilcoxon). The relations between parameters were assessed by Spearman’s rank correlation coefficient and by linear regression analysis. The linear regression equations were calculated as *y* = *βx* + *a* (*y*, dependent variable; *β*, regression coefficient; *x*, independent variable; *a*, constant term). We presented only those equations where both regression coefficient and constant term were statistically significant. Statistical analysis was performed using the package Statistica ver. 9.0. A *p* value < 0.05 was considered significant.

## Results

### Hsp27

**Fig. 1:**
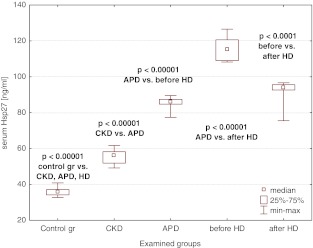
Serum Hsp27 concentrations in the examined groups

Hsp27 median values were significantly increased in CKD stage 5 children and in all dialyzed patients when compared to controls (Fig. 1). The concentrations in pre-dialysis subjects were significantly lower than in those on dialysis, irrespective of the modality. Although a single HD session decreased the concentrations of Hsp27, they were elevated vs. those in APD children both before and after a single HD session (Fig. 1).

### sFas, sFasL, MMP-7, and TIMP-1

**Fig. 2:**
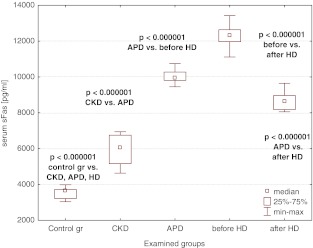
Serum sFas concentrations in the examined groups

**Fig. 3:**
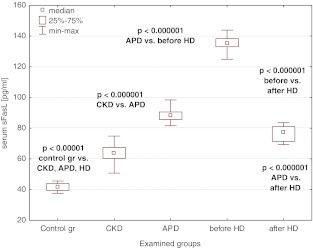
Serum sFasL concentrations in the examined groups

**Fig. 4:**
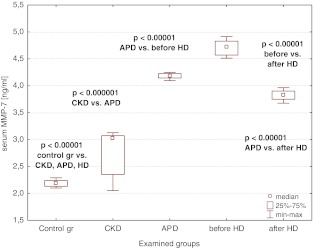
Serum MMP-7 concentrations in the examined groups

**Fig. 5:**
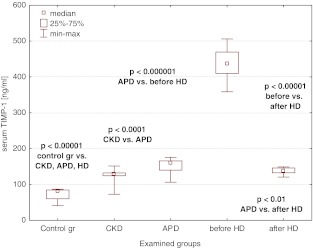
Serum TIMP-1 concentrations in the examined groups

The median values of sFas, sFasL, MMP-7, and TIMP-1 were increased in the CKD population when compared to the controls and rose on dialysis, being the highest in patients on hemodialysis (Figs. 2, 3, 4, 5). A single HD session diminished the concentrations of sFas, sFasL, MMP-7, and TIMP-1 to the values lower than those in APD patients, yet they remained higher than the levels in CKD patients (Figs. 2, 3, 4, 5).

### hsCRP, IL-4, sE-selectin

hsCRP levels did not differ between examined groups. IL-4 concentrations, although increased in all pre-dialysis and dialyzed children, failed to distinguish between CKD and different modalities. sE-selectin values rose with dialysis commencement and were higher in HD subjects than in the APD ones. However, a single HD session had no impact on any of the abovementioned parameters.

### Linear regression analysis

Since there was no difference in results of regression analysis between APD and HD patients, we decided to treat them as a whole in order to get more reliable data from a bigger group.

Consequently, sFas, sFasL, MMP-7, and TIMP-1 predicted most accurately (*R* ^2^ ≥ 0.8) the values of Hsp27 in the group of dialyzed patients (Table 1). Contrarily, in the CKD population they did not show a sufficiently predictive value.

**Table 1:** The linear regression analysis of assessed parameters in CKD children and in all patients on dialysis (APD + HD)

Dependent variable	Independent variable	Regression coefficient β	Constant term	Coefficient of determination *R* ^2^	*p*
CKD patients					
Hsp27 [ng/ml]	sFas [pg/ml]	0.003	39.67	0.29	0.03
	sFasL [pg/ml]	0.38	31.12	0.36	0.007
	MMP-7 [ng/ml]	2.12	49.41	0.009	0.28
	TIMP-1 [ng/ml]	0.08	45.56	0.12	0.08
	hsCRP [mg/l]	−1.77	56.47	0.13	0.06
	IL-4 [pg/ml]	−0.02	56.14	0.002	0.81
	sE-selectin [pg/ml]	0.08	49.84	0.11	0.09
APD + HD patients					
Hsp27 [ng/ml]	sFas [pg/ml]	0.01	−24.17	0.80	0.002
	sFasL [pg/ml]	0.62	31.47	0.89	0.000001
	MMP-7 [ng/ml]	50.05	−122.58	0.84	0.000001
	TIMP-1 [ng/ml]	0.11	70. 47	0.89	0.000001
	hsCRP [mg/l]	0.21	97.58	0.01	0.48
	IL-4 [pg/ml]	0.34	84.58	0.06	0.42
	sE-selectin [pg/ml]	0.06	93.59	0.004	0.69

No significant associations between Hsp27 and inflammation/endothelial dysfunction markers (hsCRP, IL-4, sE-selectin) were observed. No correlations with selected parameters of dialysis adequacy, such as Kt/V, hemoglobin, albumin, urea, calcium, phosphate, or parathormone levels were noticed either.

## Discussion

Our study describes for the first time the disturbed Hsp27 concentrations, and the potential role of sFas/sFasL, MMP-7 and TIMP-1 in their regulation, in children with chronic kidney disease treated conservatively and on chronic dialysis.

Hsp27 serum levels were increased in our pre-dialysis patients, which is concordant with previous observations in adults, suggesting the progressive character of those changes and their dependence on the aggravation of renal failure (Lebherz-Eichinger et al. 2012). CKD has a multifactorial stress background, conditioned by uremic toxicity, chronic inflammation, and increased oxidative stress. The progression of kidney disease is also connected with decreasing medullary perfusion, and serum Hsp27 has recently been connected with this parameter (Marquez et al. 2012). Therefore, all abovementioned factors may account for cell disintegration and subsequent enhancement of extracellular Hsp concentrations. Moreover, the effect of Hsp elevation may be strengthened by the molecule accumulation due to CKD progression, especially when it is confirmed by an inverse correlation with eGFR (Lebherz-Eichinger et al. 2012).

Our main interest was focused on the comparison of children with CKD stage 5, not on dialysis yet, with those who have already started the renal replacement therapy. Therefore, we analyzed the patients with comparable eGFR values, to eliminate its impact on possible Hsp27 increase concomitant with the dialysis commencement. Indeed, further Hsp27 elevation, appearing along with the dialysis initiation and present in the case of both modalities, strongly suggests that renal replacement therapy per se may create a stressful condition, aggravating cell damage intensity. That increase was more evident in patients on hemodialysis than in those who were dialyzed peritoneally, showing the stronger destructive potential of HD when confronted with APD. Another explanation for APD–HD discrepancy might be the difference in adequacy of both methods. Clinical data show that such criteria as metabolic and anemia control, are better fulfilled in APD children than in the HD ones. One of the reasons is the fact that every night exchanges in APD cause less fluctuations in uremic toxin concentrations than HD performed only three times a week. It also gives rise to a suggestion that the increase in hemodialysis frequency would diminish sinusoidal changes in parameter concentrations, approximating the continuous schedule of nightly peritoneal dialysis.

When a single HD session was analyzed, the concentrations of Hsp27, sFas, sFasL, MMP-7, and TIMP-1 were significantly lower after dialysis than before it. Among probable explanations of post-dialytic decrease, there are such as adsorption on the dialyzer surface or formation of various complexes: sFas–sFasL, MMP-7–TIMP-1, or Hsp27–anti-Hsp27 binding. However, the presence of anti-Hsp27 antibodies has only been proved in the patients with coronary syndromes (Shams et al. 2008). Moreover, the diminished post-dialysis Hsp27 values were still higher than those in children on peritoneal dialysis. Contrarily, other markers showed a decreasing tendency in post-dialysis concentrations vs. values in APD patients. Therefore, the pattern of Hsp27 behavior during hemodialysis session differs from that characteristic for sFas/sFasL, MMP-7, or TIMP-1. Since molecular masses of analyzed molecules are not largely discrepant (27–50 kDa), the difference in filtration is rather negligible. Thus, the release of extracellular Hsp27 due to the contact of cells with the dialyzer membrane, not compensated by the molecule elimination, seems possible.

Such discrepancy in favor of APD seems of paramount significance in children because in this population peritoneal dialysis is a method of choice. The only available comparative data concerning Hsp27 in dialyzed subjects come from a single study, where the correlation between serum Hsp27 and carotid intima-media thickness was noticed in adults on hemodialysis (Mohammadpour et al. 2011). However, there was no reference control group, so the potential serum Hsp27 increases in chronically dialyzed patients could not be proven. The patients on peritoneal dialysis were not taken into account either. Therefore, to the best of our knowledge, this study is the first analyzing serum Hsp27 both in hemodialysis and peritoneal dialysis patients, in comparison with pre-dialysis CKD subjects and control group.

The interesting finding was the correlations between Hsp27 and its potential regulators, strengthened by the results of linear regression analysis. All examined parameters (MMP-7, TIMP-1, sFas, sFasL) predicted Hsp27 concentrations in pediatric patients on dialysis. Moreover, our previous investigation has revealed that MMPs and TIMPs may well predict sFas/sFasL in dialyzed children (Musiał and Zwolińska 2011). MMP-7 engagement in sFasL regulation has also been proven by others and seems probable for sFas too (Vargo-Gogola et al. 2002; Musiał and Zwolińska 2011; Williams et al. 2010). Additionally, TIMP-1 anti-apoptotic activity, independent of its inhibitory action towards MMPs, has been described recently (Guo et al. 2006). The role of sFas/sFasL system in apoptosis regulation was analyzed within the aspect of dialysis as well (Musiał and Zwolińska 2011).

All together, these connections strongly suggest the engagement of evaluated parameters in tissue homeostasis and regeneration following the cell damage. In the case of MMPs and TIMPs, such activity results from their functioning as proteolytic enzymes, degrading and remodeling the matrix components. The sFas/sFasL impact on apoptosis is also the tool for controlling tissue destruction and restoration. Thus, the fact that MMPs, TIMPs, sFas, and sFasL are Hsp27 predictors in dialyzed patients, suggests the potential role of Hsp27 as a marker of cell damage seen in patients with chronic kidney disease.

However, some differences require careful attention. Both MMP-7 and TIMP-1 turned out to be useful predictors of Hsp27 concentrations in patients on dialysis, whereas no such link was observed in the case of pre-dialysis children with CKD stage 5. The same results concerned sFas and sFasL—they predicted very accurately Hsp27 concentrations in dialyzed subjects, but were ineffective as predictors of Hsp27 in the population of pre-dialysis CKD children. Such discrepancy may mean that factors triggering Hsp27 increase vary between those populations and uremic toxicity plays a paramount role in pre-dialysis patients, whereas in subjects on dialysis the therapy itself, with its bio-incompatibility and inadequacy above all, is able to trigger and aggravate dialysis-related stress reactions. Together with the discrepancies between HD and APD, favoring the latter, Hsp27 serum levels create a new quality in assessing biocompatibility of dialysis modalities. Surely, this theory needs to be verified in the course of future investigation performed on a bigger group of patients.

Finally, the hypothetical chain of reactions can be built, starting with Hsp27 overexpression due to stress conditions characteristic for chronic kidney disease. The next step would be the stimulation of pro-inflammatory cytokines and NF-κB (Parcellier et al. 2003), triggering MMP and TIMP overactivity that in turn determines sFas/sFasL release. The latter would control the intensity of apoptosis, thus influencing the process of tissue damage followed by restoration. Consequently, the amount of extracellular Hsp27 that is released could serve as an easily accessible universal marker of a degree of cell damage due to CKD-related confounders such as uremic toxicity or bio-incompatibility of materials used for dialysis procedures. Of note, its additional value can be derived from the fact that it is independent of potential cofactors influencing CKD, such as inflammation or endothelial damage. In this respect, all results of regression analysis would be the proof of serum Hsp27 applicability in assessing the degree of cell damage resulting from bio-incompatibility in children on chronic dialysis.

## Conclusions

Children with chronic kidney disease are prone to Hsp27 dysfunction, aggravated by the dialysis commencement and more pronounced in patients on hemodialysis. Correlations between Hsp27 and examined parameters suggest the potential role for Hsp27 as a marker of bio-incompatibility-related cell damage in the pediatric population on chronic dialysis.
